# Trends in the lifetime risk of developing cancer in Great Britain: comparison of risk for those born from 1930 to 1960

**DOI:** 10.1038/bjc.2014.606

**Published:** 2015-02-03

**Authors:** A S Ahmad, N Ormiston-Smith, P D Sasieni

**Affiliations:** 1 Queen Mary University of London, Centre for Cancer Prevention, Wolfson Institute of Preventive Medicine, Charterhouse Square, London EC1M 6BQ, UK; 2 Cancer Research UK, Head of Statistical Information, Angel Building, 407 St John Street, London EC1V 4AD, UK

**Keywords:** lifetime risk, cancer incidence rates, cancer mortality rates, all-causes mortality rates, APC model

## Abstract

**Background:**

Typically, lifetime risk is calculated by the period method using current risks at different ages. Here, we estimate the probability of being diagnosed with cancer for individuals born in a given year, by estimating future risks as the cohort ages.

**Methods:**

We estimated the lifetime risk of cancer in Britain separately for men and women born in each year from 1930 to 1960. We projected rates of all cancers (excluding non-melanoma skin cancer) and of all cancer deaths forwards using a flexible age-period-cohort model and backwards using age-specific extrapolation. The sensitivity of the estimated lifetime risk to the method of projection was explored.

**Results:**

The lifetime risk of cancer increased from 38.5% for men born in 1930 to 53.5% for men born in 1960. For women it increased from 36.7 to 47.5%. Results are robust to different models for projections of cancer rates.

**Conclusions:**

The lifetime risk of cancer for people born since 1960 is >50%. Over half of people who are currently adults under the age of 65 years will be diagnosed with cancer at some point in their lifetime.

What is the probability of developing cancer for someone born in a given year? The lifetime risk of developing cancer is the probability that a person will be diagnosed with cancer over the course of his or her lifetime. The lifetime risk is widely used as a popular measure of how widespread cancer is in a particular population (Cancer Research UK, 2012; American Cancer Society, 2013; National Cancer Institute, 2014). It is commonly expressed as a percentage (e.g., 25, 33 or 50%) or using odds (e.g., 1 in 4, 1 in 3 or 1 in 2). The lifetime risk of developing cancer for individuals born in 1900 is simply the proportion of that birth cohort that was diagnosed with cancer (assuming none are still alive and cancer free today). In practice, the cited lifetime risk is usually an artificial construct obtained by applying the cancer incidence and the all-cause mortality rates at different ages in a particular year as if they were to apply to a cohort as they aged (Feuer *et al*, 1993; Wun *et al*, 1998). This method would only be a true reflection of lifetime risk, if age-specific cancer rates and all-cause mortality rates were stable over a long time. Calculating the lifetime risk for an actual cohort is more complicated because it requires an estimate of incidence and mortality for the whole lifetime of individuals in the cohort (Campbell *et al*, 1994). Here, we combine actual rates with projected rates in order to estimate the lifetime risk for men and women born in 1930 and repeat the approach for each birth cohort from 1931 to 1960.

## Subjects and methods

All-causes mortality rates *m*_*i*_(*y*) (per 100 000 person years) (historic and projected from 2010 based) for the period ‘*y*' 1951–2012 and projected for 2013–2060 by age ‘*i*' (in 5-year bands: 0–4, 5–9, ... 80–84 and 85+) and sex were downloaded from the Office for National Statistics website (Office of National Statistics, 2012). For the calculations here we used rates from 1951 for earlier years (1930–1950).

National population estimates *N*_*i*_(*y*), as well as numbers of all cancer excluding non-melanoma skin cancer (ICD-10 codes: C00-C97 excluding C44) diagnoses *R*_*i*_(*y*) (for *y*=1975, ..., 2009) and numbers of all cancer deaths *D*_*i*_(*y*) (for *y*=1971, ... ,2009), by the 5-year age group and sex, were provided by Cancer Research UK. They in turn received the data from ONS and the relevant national cancer registries.

The lifetime risk of cancer is estimated from the rates of cancer (incidence) and all-cause mortality. The basic idea is that at each age there is a chance of being diagnosed with cancer and a chance of dying. When the cancer incidence rates include second primaries, it is necessary to consider the mortality from causes other than cancer (Sasieni *et al*, 2011). The lifetime risk is calculated taking into account the competing risk (for someone who has never had cancer) of being diagnosed with cancer and death (from something other than cancer). At any age, an individual can get cancer for the first time, can die from something other than cancer or can live without cancer until their next birthday. In Appendix 1, we provide formulae for the calculation of the lifetime risk from the rate of cancer incidence, all-cause mortality and cancer mortality. The (more usual) period estimate of lifetime risk uses rates from a give year. The cohort estimate uses rates for individuals born in a given year. Thus, the 1950 cohort will use rates from 1970 for age 20 years and from 2000 for age 50 years.

We also estimate the cumulative risk of cancer (in a cohort) up to different ages. Informally, the cumulative risk to age 80 years can be thought of as the probability of being diagnosed with cancer before the age of 80 years, assuming that there are no competing causes of death. That is, the risk in someone who does not die of something else before the age of 80 years. Hence, the cumulative risk of cancer to age 65 years will be very similar to the lifetime risk curtailed at the age of 65 years, but the cumulative risk to age of 100 years will be substantially greater than the lifetime risk (as only a small proportion of people live to 100 years). The advantage of the cumulative risk of cancer up to age of 85 years (say) is that it does not depend on all-cause mortality rates. Thus, an increase in longevity will lead to an increase in the lifetime risk of cancer, but will have no effect on the cumulative risk.

In order to estimate the rates for years for which there are no data (i.e., future years and for years before data exist), it is necessary to model the existing rates and extrapolate. Full details are provided in Appendix 2. Briefly, cancer incidence *r*_*i*_(*y*) and mortality *d*_*i*_(*y*) rates were modelled using age-period-cohort (APC) models and extrapolated out to the year 2040 using the statistical method that was published by Mistry *et al* (2011) (using the Stata apcspline command (Sasieni, 2012) with default options), in order to obtain projected rates. Age-specific rates were taken to be constant beyond 2040. Rates for 1975 were used for all previous years (1930–1974). Similarly, mortality rates for 1930–1970 were filled in with 1971 data. Interpolation within each 5-year age group was done by assuming that rates were constant within the age group, hence all cancer incidence and mortality rate data were available for those born from 1930 to 1960 by sex for ages 0–99 years.

### Sensitivity analysis

A factorial-design sensitivity analysis was performed to investigate the effect of various assumptions and model parameters on the estimated lifetime risk. In particular, we varied the link function in the generalised linear model, the extent to which the linear drift is attenuated over time, the number of knots in the splines used for the age, period and cohort effects in the model and the method used for estimating rates before 1975. Exponential and power-5 link functions were used. In addition to moderate attenuation (75% over 17 years), we considered no attenuation and rapid attenuation (75% over 6 years). As an alternative to using 1975 incidence rates for all earlier years, we used the model fits for 1960–1974 and similarly for 1960–1970 for cancer mortality rates. In this sensitivity analysis, back projected rates for 1960 were used for all previous years (1930–1960).

## Results

**Figure 1:**
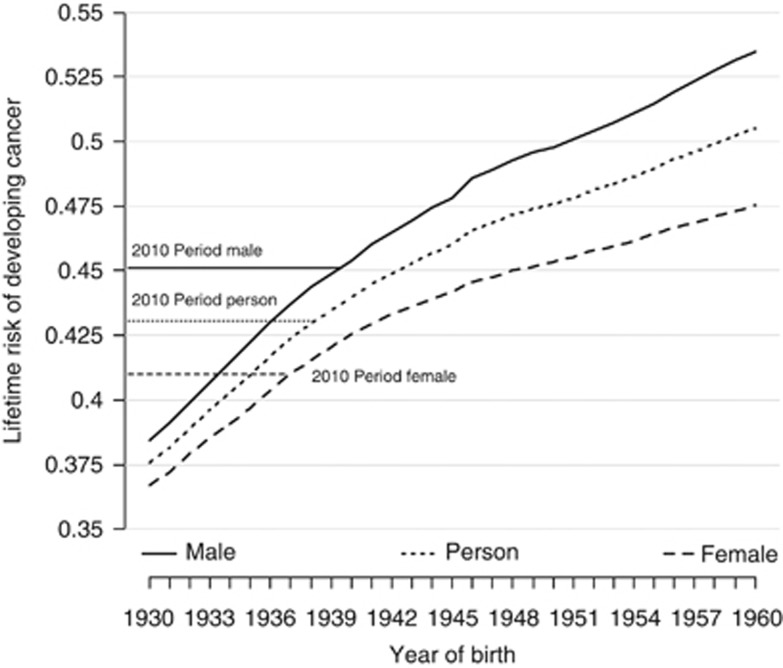
Estimated cohort lifetime risk for 1930–1960 year of birth with results from the period for 2010 lifetime risk by sex for UK population.

The estimated lifetime risks of developing cancer for men and women born in different years are presented in Table 1 and graphically in Figure 1.

The estimated lifetime risk of cancer for both men and women born in 1930 was 38.5% in men and 36.7% in women (Table 1). Over 30 years, the risks are projected to increase substantially in both sexes and the gap between sexes is predicted to increase. For men born in 1960, the lifetime risk is estimated to be 53.5% compared with 47.5% for women born in 1960. Table 2 presents the cumulative risk of cancer up to the 85th birthday (ages 0–84 years). For men born in 1930, the lifetime risk is considerably less than the cumulative risk 0–84, whereas for those born in 1960 the lifetime risk is greater: they are very similar for men born in the late 1940s. In women, the difference in risks for those born in 1930 is only slight, whereas the lifetime risk is much greater than the cumulative risk 0–84 years for those born in 1960. For both men and women, the increase in the cumulative risk over 31 years is considerably less than the increase in lifetime risk.

Figure 1 compares the results of the cohort estimates to the period estimate using rates from 2010. One can see that the 2010 period estimate of lifetime risk is similar to the cohort estimate for the 1939 and 1937 birth cohorts for men and women, respectively. Note that in 2010 men and women born in these years would have been 71 and 73 years, respectively.

By comparing Tables 1 and 2 one may infer that the main reason for the substantial increase in lifetime risk is the increasing life expectancy rather than increasing cancer incidence rates. The cumulative risk of cancer up to the age of 84 years does not take account of competing causes of death. It increased by <4%, in absolute terms, for those born in 1960 birth cohort compared with the 1930 birth cohort. By contrast, the lifetime risk in men increased by 15%.

**Figure 2:**
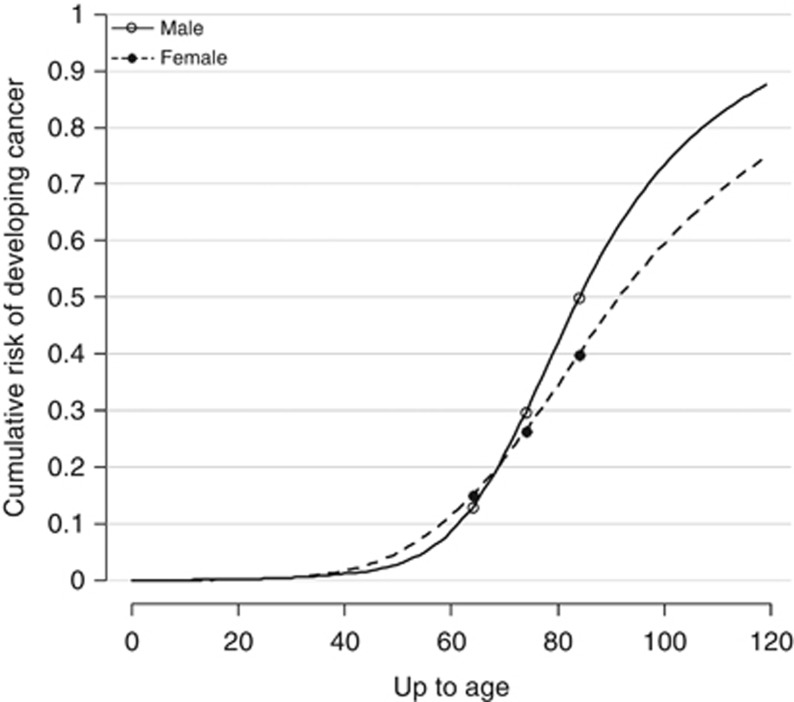
Estimated cumulative risk for 1960 cohort.

Figure 2 presents the estimated cumulative risk of cancer for the 1960 birth cohort as it ages (i.e., the cumulative risk from 0 to age *x*, for *x* between 0 and 120 years). The cumulative risk rises rapidly after the age of 65 years. Indeed, it can be seen that the cumulative risk up to age 70 years is less than half the lifetime risk. In other words, over half of the lifetime risk of cancer for the 1960 birth cohort comes from cancer diagnosed beyond the age of 70 years. The cumulative risk for men is 12.9, 29.6 and 49.8% for ages 0–64, 0–74 and 0–84 years, respectively. For women, the cumulative risk is 15.1, 26.4 and 39.9%, respectively.

It is interesting to speculate whether if people lived long enough virtually everyone would get cancer. The cumulative risk to age 120 years for men is nearly 90% but it is just over 70% for women. Thus, it would seem that virtually all men would get cancer if they did not die of other causes first. But in women, whereas the majority of women would get cancer if they did not die of other causes first, a substantial minority would not.

**Figure 3:**
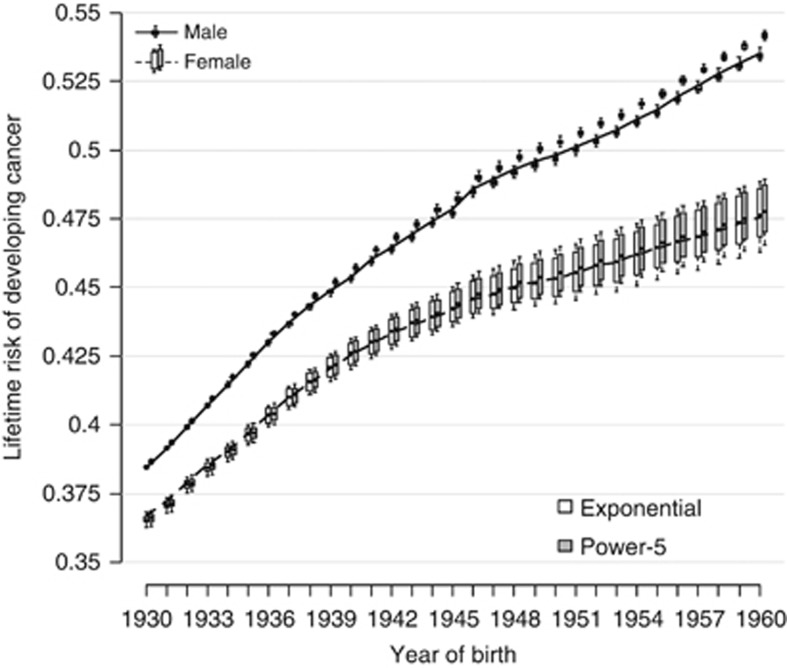
**Estimated cohort lifetime risk for 1930–1960 year of birth with results from the sensitivity analysis and period for 2010 lifetime risk by sex for UK population.** Note that for males (and females) the line goes through the exponential models estimates.

Figure 3 shows, for each year of birth, the results of the sensitivity analysis on the estimated lifetime risk. Each boxplot presents 54 estimated values of the lifetime risk for a particular sex-specific birth cohort. The variation in risk over a time is much greater than the variation in risk due to different methods of projection. All models estimate the lifetime risk of the 1960 male birth cohort to be over 50%. The sensitivity to model parameters is slightly greater for females, but the estimates are virtually all with ±1% of those presented in Figure 1.

## Discussion

The lifetime risk of cancer for men born since 1950 is >50%. For women it is slightly less. The lifetime risk for men born in 1960 is much greater than for men born in 1930: it increased from 38.5 to 53.5% or by a factor of 1.39 and we would expect this increase to continue into the future. Much of this increased risk is due to increasing longevity: the cumulative risk until the age of 84 years was 46.6% for men born in 1930 and it has increased by a factor of just 1.07% to 49.8% for men born in 1960. Similar increases are seen in women, but the impact of increasing longevity is less dramatic—lifetime risk increases by a factor of 1.30, whereas the cumulative risk up to the age of 84 years increases by 1.11 (1960 birth cohort compared with that of 1930).

Men born since 1950 have >1 in 2 chance of being diagnosed with cancer at some point in their lifetime. It is noticeable that the lifetime risk increased more rapidly for cohorts between 1930 and 1940 than between 1950 and 1960 (Figure 1). To the extent that we have assumed that cancer rates are constant after age 85 years, we could have underestimated the lifetime risk in the latter birth cohorts many of who will live beyond the age of 85 years.

Beyond the effect of increasing longevity, increasing age-specific rates of cancer have also had an impact on the increasing lifetime risk of cancer. Whereas smoking-related cancers have become less common in men, other cancers have become more common.

In women, breast and lung cancers have increased substantially since the mid 1970's. The increase in breast cancer is related to lifestyle changes, such as increasing obesity (Renehan *et al*, 2008), women having fewer children, at later ages (Ewertz *et al*, 1990) and breast screening detecting more breast cancers at younger ages.

In men, there has been an increase in the incidence of prostate and bowel cancer. A large proportion of the increase in prostate cancer diagnoses has been caused by prostate-specific antigen testing detecting cancers that would not otherwise have been diagnosed (Bray *et al*, 2010). The increase in bowel cancer rates is thought to be related to an increase in red meat consumption and obesity (Center *et al*, 2009).

There are some limitations of the available data. We do not have incidence, mortality or population data to cover the entire lifetime of the cohorts studied. It should although be noted that someone born in 1930 would in 2012 either have died already or be 84 years old, so their lifetime risk of cancer requires little forward extrapolation. Although we have no incidence data before 1971, the cumulative risk of cancer to age 40 years (someone born in 1930 would have been 41 years in 1971) is small and variations in cancer rates in children, teenagers and young adults will have little impact on the eventual lifetime risk. By contrast, someone born in 1960 is unlikely to have died and would, in 2012, be 51 years so most of their lifetime risk is extrapolated.

The sensitivity analysis (Figure 3) shows that although the method of estimating future (and past) rates affects the final answer, the variation in lifetime risk resulting from choice of model parameters is small compared with the variation over 30 years of birth cohorts. The results are therefore reasonably robust.

The results of this analysis should enhance public health messages and improve resource planning for both commissioners and providers of healthcare in the UK. It may also assist clinicians and patients to weigh the lifetime risk of developing cancer versus other challenging health risks. Whereas the results of this analysis are specific to the British population, the methodology can be applied to other populations.

## Figures and Tables

**Table 1:** Estimated lifetime of developing cancer by year of birth and sex for age group 0–99 years

**Year of birth**	**1930**	**1935**	**1940**	**1945**	**1950**	**1955**	**1960**
Male (%)	38.5	42.2	45.4	47.8	49.8	51.5	53.5
Female (%)	36.7	39.7	42.6	44.2	45.3	46.4	47.5

**Table 2:** Estimated cumulative risk of developing cancer by year of birth and sex for the age group 0–84 years

**Year of birth**	**1930**	**1935**	**1940**	**1945**	**1950**	**1955**	**1960**
Male (%)	46.6	46.9	47.7	48.0	48.6	49.2	49.8
Female (%)	36.1	36.8	38.4	39.0	39.4	39.7	39.9

**Table tbla1:** Parameters used in the modelling

	Baseline	Other values
Link	Exponential	Power-5
Damping	0.92	0.85, 0.96
Knots: age	6	4, 8
Knots: period	5	
Knots: cohort	3	2, 5

## Appendix 1

**Calculation of the lifetime risk of cancer by the cohort method**

For the period ‘*y*' and age ‘*i*': let *m*_*i*_(*y*) denote the all-causes mortality rates; *r*_*i*_(*y*) the incidence of all cancers (excluding non-melanoma skin cancer; ICD-10 codes C00-C97 excluding C44) and *d*_*i*_(*y*) the (all) cancer mortality rate.

For ease of notation we drop the argument *y* when it is constant (i.e., we write *r*_*i*_ for *r*_*i*_(*y*)).

The lifetime risk, LR can be estimated by using the following summation over all ages (see Sasieni *et al* (2011) for further details): write *λ*_*i*_=*r*_*i*_+*m*_*i*_−*d*_*i*_ for the hazard of no longer being alive and cancer free and define *l*_*i*_ to be the contribution to the lifetime risk at age *i*. Note that *l*_*i*_ is shorthand for *l*_*i*_(*y*), where *y* is the period (and the year of birth is *y*−*i*).

Let

and

where , the probability of being at risk (i.e., alive and without a previous diagnosis of cancer) at age (exactly) *i* is defined as

Note that (1−exp{−*λ*_*i*_})/*λ*_*i*_ is the expected person years at risk between *i* and *i*+1for an individual who is alive and cancer free at age *i*.

The lifetime risk for period *y* is

Similarly we can define the lifetime risk for the cohort born in *y* as

The cumulative risk up to the *f*th birthday can be estimated according to the following formula (Day, 1992):

Once again the period estimate is obtained by summing the *r*_*i*_(*y*) and the cohort estimate by summing the *r*_*i*_(*y*+*i*).

## Appendix 2

**Modelling cancer incidence rates**

We use a generalised linear model applied to the numbers of cancers and population to estimate the rates of cancer by age and year of diagnosis (and hence year of birth too). The numbers of cancers are assumed to be Poisson random variables. The link function is by default taken to be the canonical link (i.e., exponential). We also consider the power-5 link function. The effects of age, period (i.e., year of diagnosis) and cohort (i.e., year of birth) are all modelled as natural cubic splines. Knots are placed at the maximum and minimum value of each variable and internal knots are placed at evenly spaced percentiles of the data. The number of internal knots is varied (see Table). The linear trends in period and cohort are termed drift. In extrapolating the drift into the future, we assume that the linear trend is dampened by a certain proportion each year. After *k* years, we assume that the drift only increases by this proportion (the damping parameter) raised to the power k. With damping set to 0.92 (our default) the drift in the eighth year beyond the last observation is approximately half of what it is during the period of observation. The non-linear trend in period is assumed to stop after the last observation. Further details are provided in Sasieni (2012).

[Table tbla1]

## References

[bib1] American Cancer Society 2013Lifetime probability of developing or dying from cancer. Available from: : http://www.cancer.org/cancer/cancerbasics/lifetime-probability-of-developing-or-dying-from-cancer (accessed 30 July 2014).

[bib2] Bray F Lortet-Tieulent J Ferlay J Forman D Auvinen A 2010 Prostate cancer incidence and mortality trends in 37 European countries: an overview Eur J Cancer 46 (17 3040 3052 21047585 10.1016/j.ejca.2010.09.013

[bib3] Campbell MK Feuer EJ Wun LM 1994 Cohort-specific risks of developing breast cancer to age 85 in Connecticut Epidemiology 5 (3 290 296 8038242 10.1097/00001648-199405000-00006

[bib4] Cancer Research UK 2012Statistics on the risk of developing cancer. Available from: : http://www.cancerresearchuk.org/cancer-info/cancerstats/incidence/risk/statistics-on-the-risk-of-developing-cancer (accessed 30 July 2014).

[bib5] Center MM Jemal A Ward E 2009 International trends in colorectal cancer incidence rates Cancer Epidemiol Biomarkers Prev 18 (6 1688 1694 19505900 10.1158/1055-9965.EPI-09-0090

[bib6] Day NE 1992 Cancer incidence in five continents. Cumulative rate and cumulative risk IARC Sci Publ 120 862 864 1284607

[bib7] Ewertz M Duffy SW Adami HO Kvale G Lund E Meirik O Mellemgaard A Soini I Tulinius H 1990 Age at first birth, parity and risk of breast cancer: a meta-analysis of 8 studies from the Nordic countries Int J Cancer 46 (4 597 603 2145231 10.1002/ijc.2910460408

[bib8] Feuer EJ Wun LM Boring CC Flanders WD Timmel MJ Tong T 1993 The lifetime risk of developing breast cancer J Natl Cancer Inst 85 (11 892 897 8492317 10.1093/jnci/85.11.892

[bib9] Mistry M Parkin DM Ahmad AS Sasieni P 2011 Cancer incidence in the United Kingdom: projections to the year 2030 Br J Cancer 105 (11 1795 1803 22033277 10.1038/bjc.2011.430 PMC3242594

[bib10] National Cancer Institute 2014Lifetime risk. Available from: http://surveillance.cancer.gov/statistics/types/lifetime_risk.html (accessed 30 July 2014).

[bib11] Office of National Statistics 2012Historic and Projected Mortality Data (1951 to 2060) from the UK Life Tables, 2010-Based Release. Available from: : http://www.ons.gov.uk/ons/publications/re-reference-tables.html?edition=tcm%3A77-257453 (accessed 30 July 2014).

[bib12] Renehan AG Tyson M Egger M Heller RF Zwahlen M 2008 Body-mass index and incidence of cancer: a systematic review and meta-analysis of prospective observational studies Lancet 371 (9612 569 578 18280327 10.1016/S0140-6736(08)60269-X

[bib13] Sasieni PD 2012 Age-period-cohort models in Stata Stata J 12 (1 45 60

[bib14] Sasieni PD Shelton J Ormiston-Smith N Thomson CS Silcocks PB 2011 What is the lifetime risk of developing cancer?: the effect of adjusting for multiple primaries Br J Cancer 105 (3 460 465 21772332 10.1038/bjc.2011.250 PMC3172907

[bib15] Wun LM Merrill RM Feuer EJ 1998 Estimating lifetime and age-conditional probabilities of developing cancer Lifetime Data Anal 4 (2 169 186 9658774 10.1023/a:1009685507602

